# Supplementary material for: Inhibitory synaptic transmission is impaired at higher extracellular Ca2+ concentrations in Scn1a+/− mouse model of Dravet syndrome
Source: Sci Rep. 2021 May 20;11:10634. doi: 10.1038/s41598-021-90224-4 (PMC8137694; doi:10.1038/s41598-021-90224-4)
Supplement: Supplementary file 1 — Supplementary Information. [file 41598_2021_90224_MOESM1_ESM.pdf]

a

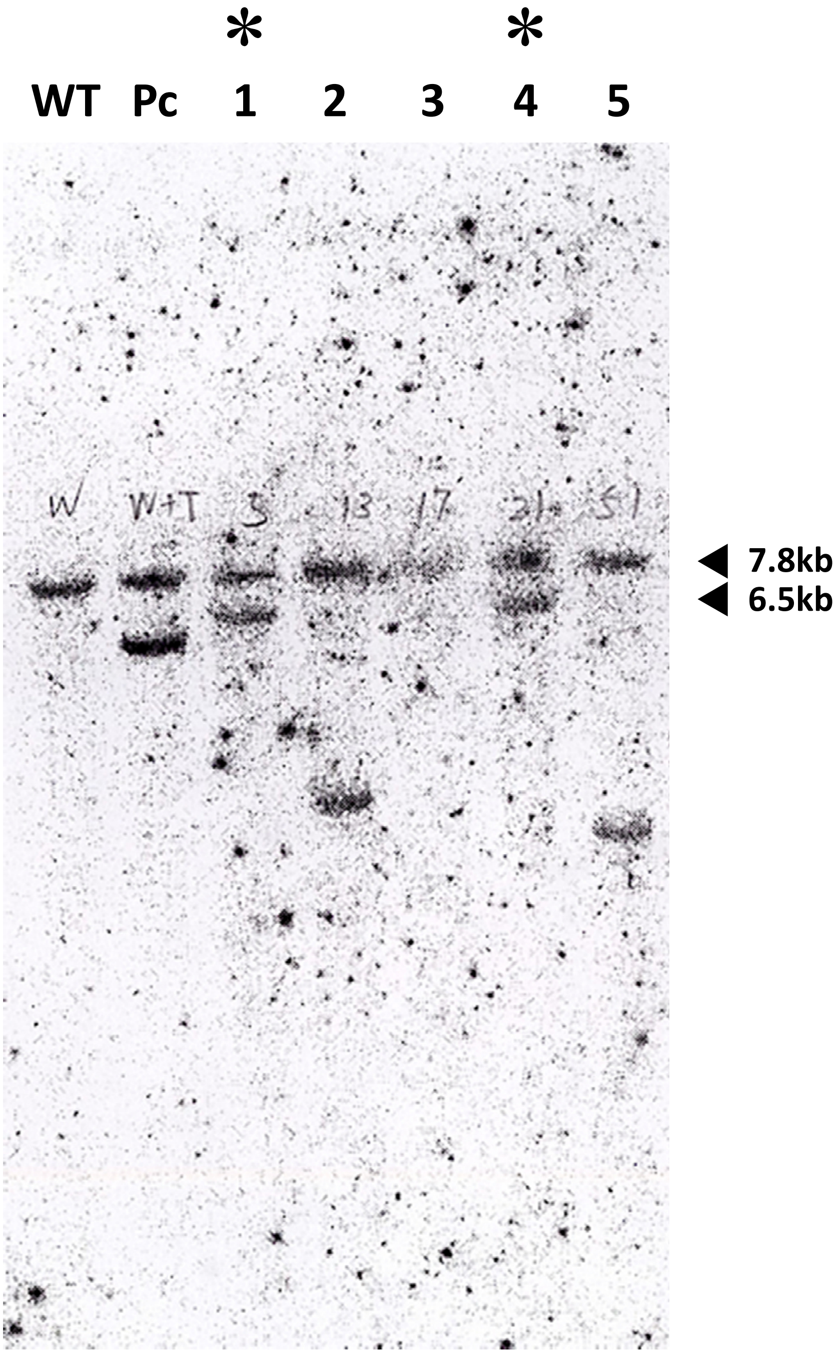

Figure S1. Raw data underlying Figure 1c.
(**a**) Full-length image of the Southern hybridization for seven candidate clones. Handwritten characters indicate the identification numbers of the samples.

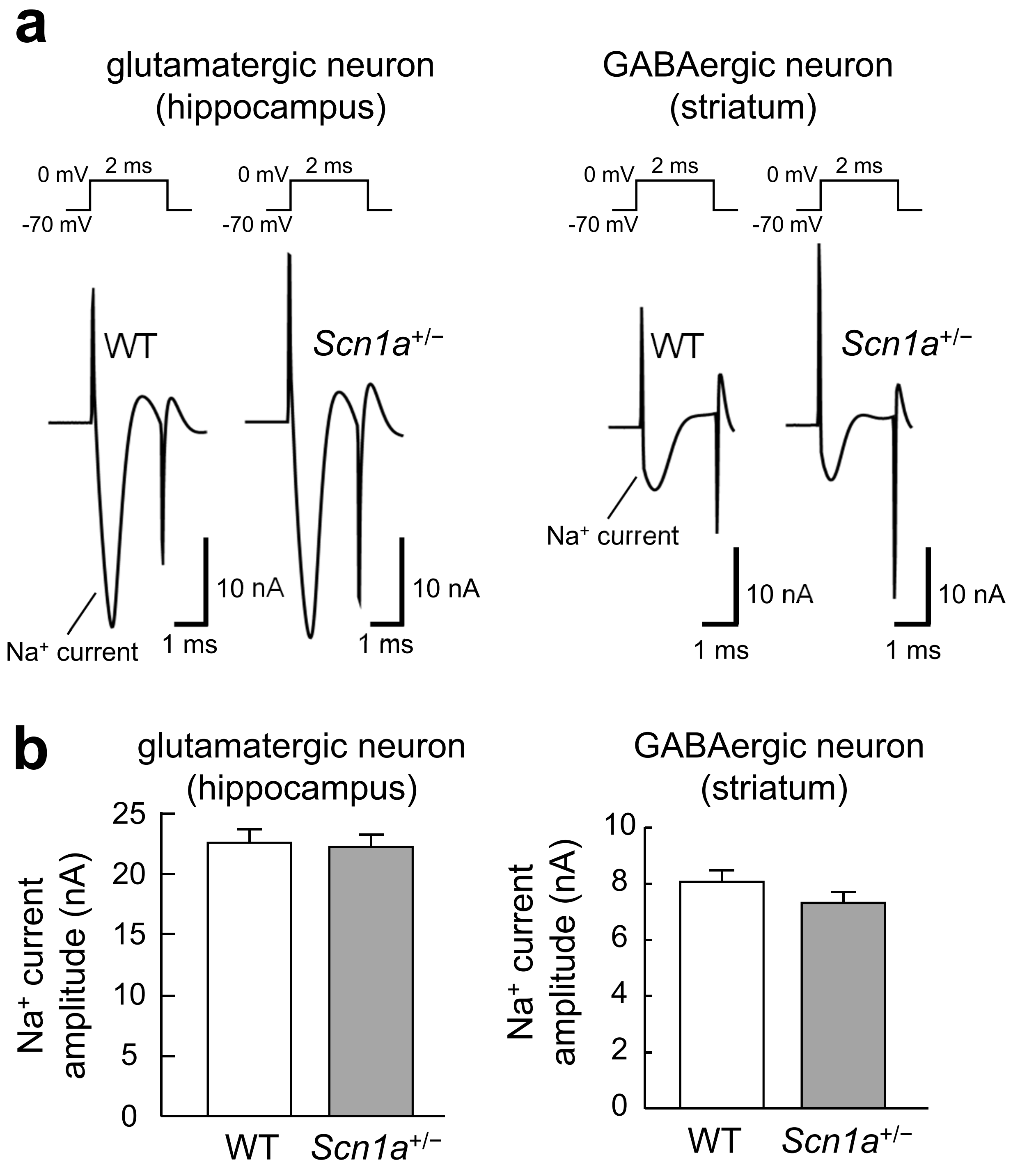

Figure S2. No change in the Na^+ current amplitude in WT and *Scn1a*$^{+/-}$ neurons of the hippocampus and striatum.

(**a**) Representative traces of Na^+ currents evoked by depolarizing pulses (-70 mV to 0 mV) via patch pipette for 2 ms under the voltage-clamp condition. Note that there is no difference in Na^+ currents between WT and *Scn1a*$^{+/-}$ neurons of the hippocampus and striatum. (**b**) Average amplitudes of the Na^+ current in WT and *Scn1a*$^{+/-}$ neurons of the hippocampus and striatum.
